# The effect of transcranial random noise stimulation (tRNS) over bilateral posterior parietal cortex on divergent and convergent thinking

**DOI:** 10.1038/s41598-020-72532-3

**Published:** 2020-09-23

**Authors:** Javier Peña, Agurne Sampedro, Naroa Ibarretxe-Bilbao, Leire Zubiaurre-Elorza, Aralar Aizpurua, Natalia Ojeda

**Affiliations:** Department of Methods and Experimental Psychology, Faculty of Psychology and Education, University of Deusto, Avda. Universidades 24, 48007 Bilbao, , Basque Country Spain

**Keywords:** Human behaviour, Problem solving

## Abstract

Creativity pervades many areas of everyday life and is considered highly relevant in several human living domains. Previous literature suggests that the posterior parietal cortex (PPC) is related to creativity. However, none of previous studies have compared the effect of transcranial random noise stimulation (tRNS) over bilateral PPC on both verbal and visual divergent thinking (DT) and Remote Associates Test (RAT) in the same experimental design. Forty healthy participants were randomly assigned to tRNS (100–500 Hz) over bilateral PPC or sham group, for 15 min and current was set at 1.5 mA. Participants’ creativity skills were assessed before and after brain stimulation with the Unusual Uses and the Picture Completion subtests from the Torrance Test of Creative Thinking and the RAT. ANCOVA (baseline scores as covariate) results indicated that tRNS group had significantly higher scores at post-test in RAT and visual originality compared to sham group. Unusual Uses, on the other hand, was not significant. Improvement in RAT suggests the involvement of PPC during via insight solution which may reflect internally directed attention that helps the recombination of remotely associated information. The improvement in visual originality dimension from DT may be due to a higher internally directed attention while reducing externally oriented attention.

## Introduction

Creativity is considered one of the most important and prevalent human accomplishments^1^ but incredibly difficult to measure and define. However, in the scientific literature there is a relative agreement that creative solutions can be defined as both potentially novel/original and useful/effective^2–4^. According to several authors, creative thinking includes two main components^1,5,6^, including convergent thinking (CT) and divergent thinking (DT). CT involves finding a single solution to a closed-ended problem by using deductive reasoning^7^. According to many authors^8–20^ the most used instrument for CT has been the Remote Associates Test (RAT)^21^. Although RAT seems to measure mainly CT, there is also evidence that it may be reflecting DT, at least to some extent^22^.

DT, on the other hand, is usually defined as the ability to form remote associations between unconnected ideas from distant categories and produce multiple alternative and original responses to an open-ended problem^6,23^. In this context, originality is argued to contain both novelty and authenticity^24^ and the research during idea generation activates an exploratory DT process^25^.

Given the importance of creativity, the interest from a neuroscientific perspective has increased during the last years^26,27^. In this context, several studies have used neuroimaging and transcranial stimulation techniques in order to investigate the neural underpinnings of creative thinking, including both CT and DT. According to a meta-analysis of transcranial stimulation^28^, most of these studies have targeted dorsolateral prefrontal cortex (DLPFC) and frontotemporal areas^8,29–34^. Transcranial direct current stimulation (tDCS)^7,31,34^ and transcranial random noise stimulation (tRNS)^35^ over the DLPFC have consistently shown an improvement in RAT.

The literature regarding DT, on the other hand, has shown more inconsistent results after frontotemporal stimulation. Anodal tDCS over the DLPFC^30^ and prefrontal cortex^32^ has been related to higher verbal DT. In contrast, various previous studies have shown a significant general verbal DT improvement (but not originality) after cathodal (but not anodal) tDCS^8,36^ and transcranial magnetic stimulation^29^ applied to the left inferior frontal gyrus (IFG). Similarly, 10 Hz Transcranial Alternating Current Stimulation (tACS) over the DLPFC^37,38^ has been linked to an improvement in verbal DT (more precisely with ideational verbal fluency but not in originality)^38^ and a composite of visual DT (but not originality)^37^. Finally, Chi and Snyder^39,40^ showed that the inhibition of the anterior temporal lobe produced an enhancement in insight problem-solving tasks (matchstick arithmetic problems and the nine dot problem).

Even though there is not a completely consistent pattern of results, all these previous studies suggest that both DLPFC and fronto-temporal areas of the brain are more related to both verbal CT and DT (especially to verbal fluency dimension from DT), but not so much to originality.

On the other hand, the role of parietal cortex has also been related to creativity by several neuroimaging^25,41–50^ and non-invasive brain stimulation studies^1,7,51,52^, based mainly on its role as part of the default-mode network^50^. One of the most replicated findings in the literature is the strong synchronization of EEG alpha activity over PPC during performance on mainly verbal DT tasks^25,46,48,53,54^.Although less studied, EEG alpha power over PPC has also been related to visual DT^55^. Since the parietal alpha synchronization is suggested to reflect a higher internally directed attention while reducing externally oriented attention^56^, according to previous authors^57^ it may indicate that “divergent thinkers are better able to continually exclude interfering external stimuli for the sake of generating creative responses”. Additional evidence for the role of PPC on DT comes from studies using functional magnetic resonance imaging (fMRI) also suggests that PPC is related to DT^41,58^.

A closer look at these studies on DT suggests that PPC involvement in creativity may be more related to the originality dimension of DT rather than fluency^45,46,59,60^. On the contrary, as previously mentioned, prefrontal areas seem to be more related to fluency dimension of DT^8,32,37,38^.

Similar to the relationship between DT and PPC, RAT or Compound Remote Associates scores have also been related to alpha power in PPC studies when participants generated solutions with insight^61,62^. According to some authors^25^, a possible explanation for the finding of alpha power increase in PPC during insightful solutions may be interpreted as a suppression of sensory information, thus favoring the combination of remotely associated semantic information^25^. Studies that included fMRI^63,64^ suggest that along with frontal brain regions (including DLPFC, ventrolateral PFC, medial PFC and IFG), PPC was involved in Compound Remote Associates Task performance. There are only two studies that used tDCS over the PPC^1,7^ and their the results are in line with the evidence from neuroimaging studies. In the study carried out by Zmigrod et al.^7^ they found that anodal stimulation of both left and right PPC produced an increase of insight type solutions to RAT. However, the same study did not show a significant improvement in verbal DT. They did not include any visual DT task, so we cannot conclude if the lack of improvement found in verbal DT would be also true for visual DT. Similarly, Pick and Lavidor^1^ found an improvement in RAT after anodal bilateral stimulation of PPC. In another study using tDCS, Ghavanati et al.^51^ found that anodal stimulation over the right PPC produced more unique and novel figures than anodal stimulation over the DLPFC and sham groups in a figural fluency task. These unique and novel figures may be considered an originality measure, although it is not directly comparable to other originality tasks from visual DT (such as Torrance Test of Creative Thinking^65^) used in the neuroimaging studies previously mentioned.

However, as far as the authors are aware, none of the previous studies targeting the PPC has examined its role on visual DT tasks.

On the other hand, most previous studies that investigated creative thinking through transcranial stimulation have used tDCS. TRNS is a more recent form of transcranial electrical stimulation of random noise to modulate cortical plasticity, especially when using high frequencies^66^ even though the underlying mechanisms are not yet completely understood. There are two main suggested mechanisms of tRNS effects, including the increase of neuronal excitability via stochastic resonance (whereby weak neural signal detection in the central nervous system is enhanced when ‘noise’ is added^67^) and the repetitive opening of NaC channels^68^. Although more research is needed, previous studies have suggested some advantages that tRNS may show compared to tDCS and tACS. For example, the effect of tRNS may be larger than tDCS in visual perceptual learning tasks^66^ and have a greater long-term effect^69,70^. Moreover, tRNS has been proposed to be more tolerable than tDCS regarding possible adverse effects, such as irritation and burning^71^. Finally, a meta-analysis^72^ indicated that the effect of tRNS on language and mathematics was stronger than that of tDCS.

Therefore, based on previous studies that used tDCS over PPC^1,7^, our first hypothesis was that participants receiving tRNS over bilateral PPC would improve their CT scores (RAT) compared to sham.

Considering the impact of tDCS over PPC on figural creativity^51^ but not in verbal DT^7^, our second hypothesis was that tRNS would improve more in visual DT than in verbal DT. Finally, given that the literature suggests that prefrontal brain regions are more related to the fluency dimension of DT^8,32,37,38^ whereas PPC is more related to the originality dimension^45,46,59,60^, we expect to find a higher positive effect of tRNS over PPC on the originality dimension of DT compared to fluency.

## Results

### Baseline characteristics of tRNS and sham groups

Baseline characteristics of the groups are shown in Table 1. There were no significant differences in age, years of education, gender, number of hours slept, number of stimulants (tea, coffee or similar), and handedness (Edinburgh Handedness Inventory). We asked if the number of stimulant drinks ingested and number of hours slept was more than usual, less than usual or as usual at the time of assessment. There were no significant differences between groups in any of the proportion of these responses regarding stimulant drinks (X^2^ = 0.01, *p* = 0.924) and sleeping condition (X^2^ = 0.58, *p* = 0.748).

**Table 1:** Participant characteristics at baseline.

	tRNS group		Sham group		Statistic	*p*
	Mean (SD)	Range	Mean (SD)	Range		
Age	21.55 (1.79)	9	22.15 (4.35)	21	*F* = 0.33	0.572
Years of education	15.05 (2.19)	9	15.11 (2.35)	7	*F* = 0.01	0.940
Gender: n (%)						
Females	16 (80.0%)		15 (75.0%)		X^2^ = 0.14	0.715
Male	4 (20.0%)		5 (25.0%)			
Number of slept hours	7.43 (1.52)	6	7.53 (1.23)	4.5	*F* = 0.05	0.820
Edinburgh handedness	53.49 (57.01)	185.7	68.64 (28.46)	125	*F* = 1.13	0.294
Number of stimulants	0.65 (0.81)	3	0.55 (0.60)	2	*F* = 0.20	0.661

tRNS, transcranial random noise stimulation; SD, standard deviation.

### Effect of brain stimulation on convergent thinking, verbal divergent thinking and visual divergent thinking

Table 2 shows baseline and post-treatment scores for all creative sub-domains. In Table 3 we show marginal means (with baseline scores as covariates) of RAT, visual DT and verbal DT scores after brain stimulation. ANCOVA results indicated that there were significant differences between tRNS and sham groups in RAT, suggesting a higher number of correct responses after stimulation in the tRNS group compared to sham. Results regarding visual DT scores and verbal DT, on the other hand, were not statistically significant.

**Table 2:** Creativity scores in the active tRNS and the sham group at pre- and post-intervention.

	tRNS group	Sham group
	Mean (SD)	Mean (SD)
**RAT**		
Pre	5.50 (2.94)	6.10 (2.22)
Post	7.80 (4.44)	5.60 (2.11)
**Figural fluency**		
Pre	5.00 (1.83)	5.20 (1.85)
Post	5.55 (2.21)	5.75 (2.09)
**Figural originality**		
Pre	1.40 (1.18)	2.10 (1.07)
Post	2.60 (1.42)	1.70 (1.21)
**Figural flexibility**		
Pre	4.35 (1.49)	4.45 (1.79)
Post	4.75 (1.88)	4.65 (1.53)
**UU fluency**		
Pre	6.55 (2.62)	7.35 (2.75)
Post	6.95 (3.31)	6.85 (2.47)
**UU originality**		
Pre	4.50 (2.83)	4.10 (3.05)
Post	4.30 (2.27)	4.47 (1.92)
**UU flexibility**		
Pre	4.90 (2.36)	5.35 (2.30)
Post	4.80 (2.11)	5.85 (2.05)
**Visual DT**		
Pre	− 0.14 (0.83)	0.12 (0.93)
Post	0.11 (0.96)	− 0.10 (0.75)
**Verbal DT**		
Pre	− 0.08 (0.91)	0.04 (0.86)
Post	− 0.10 (0.81)	0.08 (0.87)

tRNS, transcranial random noise stimulation group; SD, standard deviation; UU, unusual uses test from the torrance test of creative thinking; Verbal DT, verbal divergent thinking composite score; Visual DT, visual divergent thinking composite score.

**Table 3:** Post-treatment marginal means in creativity scores in the active tRNS and the sham group controlling for baseline scores.

	tRNS group	Sham group	*F*	*p*	Effect size (${\mathrm{n}}_{\mathrm{p}}^{2})$
	Marginal mean (standard error)	Marginal mean (standard error)			
RAT	8.01 (0.68)	5.39 (0.68)	7.37	0.010	0.166
Visual DT	0.19 (0.14)	− 0.19 (0.14)	3.63	0.065	0.089
Verbal DT	− 0.06 (0.14)	0.04 (0.14)	0.25	0.624	0.007

tRNS, transcranial random noise stimulation; RAT, Remote Associates Test; Verbal DT, verbal divergent thinking composite score; Visual DT, visual divergent thinking composite score; ${\mathrm{n}}_{\mathrm{p}}^{2}$, eta partial squared.

A closer inspection of the visual DT subdomains revealed that only visual originality was significant (*F* = 6.91, *p* = 0.012, ${\mathrm{n}}_{\mathrm{p}}^{2}$ = 0.157). In this subdomain, scores of the tRNS group increased from 1.40 (SD = 1.18) to 2.60 (SD = 1.42), whereas the sham group changed from 2.10 (SD = 1.07) to 1.70 (SD = 1.21).

Regarding verbal DT subdomains (data not shown), none of them were significant (*F* values from 0.00 to 2.37, *p* values from 0.966 to 0.132).

### Adverse effects and blinding

None of the participants reported having experienced any significant adverse effects. There were not significant differences between real stimulation groups vs sham in the number of adverse effects assessed (*F* = 1.17, *p* = 0.285). However, the percentage of participants experiencing concentration problems was significantly higher in the experimental group compared to sham (See Table 4).

**Table 4:** Percentage of presence of adverse effects for tRNS and sham groups.

	tRNS group	Sham group	X^2^	*p*
	N (%)	N (%)		
Headache	1 (5.0%)	4 (21.1%)	2.25	0.134
Throat sore	0 (0.0%)	2 (10.5%)	2.21	0.136
Scalp pain	2 (10.0%)	1 (5.3%)	0.31	0.579
Skin tingling	11 (55.0%)	8 (42.1%)	0.65	0.421
Skin itching	10 (50.0%)	5 (26.3%)	2.30	0.129
Skin burning sensation	5 (25.0%)	2 (10.5%)	1.38	0.239
Redness of the skin	2 (10.0%)	0 (0%)	2.00	0.157
Numbness	1 (5.0%)	2 (10.5%)	0.42	0.517
Concentration problems	6 (30.0%)	1 (5.3%)	4.05	0.044
Mood change	3 (15.0%)	2 (10.5%)	0.17	0.676
Phosphenes	1 (5.0%)	0 (0%)	0.98	0.323

tRNS, transcranial random noise stimulation.

A Chi-square test was performed to test blinding of the stimulation based on participants´ report. In the stimulation group, 10.0% guessed that they had received stimulation, 25.0% placebo and 65.0% did not know. in the sham group, 10.0% guessed that they had received stimulation, 55.0% placebo, 35.0% and did not know. There were no significant differences in stimulation guess between sham and real tRNS conditions (χ^2^ = 4.05, *p* = 0.132).

## Discussion

The present study investigated the effects of increasing cortical excitability with tRNS over left and right PPC on RAT, visual DT and verbal DT tasks. We found that tRNS produced a significant and large increase in RAT scores when compared to sham. This result is consistent with previous studies that used tDCS over PPC^1,7^ and with neuroimaging studies, including EEG^61,62^ and fMRI^63,64^. Zmigrod et al.^7^ found that anodal stimulation over right and anodal over left PPC produced a higher number of insight solutions to a Compound Remote Associates Task, a similar task to RAT used in the present study. Similarly, Pick and Lavidor reported a significant increase in RAT after anodal bilateral stimulation over PPC^1^. In the present study, participants only had 165 s for the whole task, which resulted in 5.5 s per item. According to various authors^57,73^, CRA problems may be solved in at least two ways; (1) via insight (also called the “Aha moment”) that appears suddenly and with little ability to report on the processing that enabled the solution and (2) via a deliberate, trial-and-error analytical approach. Given the time constriction, participants did not have much time to think about possible responses, so we believe that the main way to obtain the solution was insight type (AHA moment) rather than an analytical approach. This hypothesis goes in line with Kounios et al.´s findings^74^, who found less alpha over the posterior cortex related to non-insightful, analytical problem-solving. Authors^74,75^ suggest that this kind of problem-solving is characterized by directing attention outward in a bottom-up way whereas insight type solution is associated with focusing attention more inwardly. However, we cannot assure it was mainly via insight since we did not ask them to report how they had obtained the correct answer directly. We did not include another condition giving more time to find the solution for RAT. Therefore, we cannot know if the effect of tRNS over PPC would have disappeared or attenuated if they had been given the chance to use other different cognitive mechanisms to solve the problem. In this context, future transcranial stimulation studies could investigate if PPC vs prefrontal brain regions improve RAT scores through different cognitive mechanisms.

Our results regarding visual DT indicate that participants receiving tRNS over PPC significantly increased the visual originality dimension of DT (but not fluency of flexibility) showing a large effect size. As far as the authors are aware, there is no previous study that examined visual DT after stimulating bilateral PPC either with tRNS or tDCS, so direct comparisons are not possible. However, this result goes in line with the previously mentioned results on neuroimaging studies^55,76^ and tDCS over PPC on figural fluency^51,52^. In the tDCS studies, the authors compared anodal stimulation over the right PPC with anodal over the left DLPFC and sham. In their first study^51^, results indicated that participants produced significantly more unique designs under anodal right PPC tDCS compared to anodal left DLPFC and sham. Similarly, in a more recent study^52^, the same authors found very similar results regarding unique and novel figures in a figural fluency task, whereas left anodal DLPFC stimulation generated more verbal fluency scores.

Taking together both RAT and visual originality results, we could speculate that the alpha power effect could reflect more internally oriented attention^53^, which may reflect selective inhibition of interfering external input^48,77,78^. This internally-directed attention could therefore facilitate the combination of remotely associated semantic information^25^ and prevent functional fixedness during the creative ideation phase of creativity^79^.However, there are other possible explanations for the role of PPC, such as its role in efficient effective memory search and retrieval that could have a positive impact on these tasks^53^, working memory for object location^80^ or spatial representation and updating^81^.

Regarding verbal DT, we did not find any significant improvement after tRNS compared to sham. These results are also consistent with the lack of significant changes after tDCS over PPC reported by the only study that used tDCS over PPC^7^. In this study, the authors assessed fluency, flexibility and elaboration but not originality. The studies that reported significant changes after transcranial stimulation in verbal DT, on the other hand, have focused on more frontal regions^8,30,32^. The lack of significant results after stimulating PPC observed in this study and the previous one^7^, altogether with significant improvement after targeting more frontal areas^7,8,30,32^ suggest that PPC might not be as strongly related to verbal DT as frontal areas. The results were non-significant even for verbal originality, a dimension more closely related to PPC as shown in different neuroimaging studies^45,46,59,60^.

Despite these interesting results, the present study has several limitations. First, we did not compare tDCS directly with tRNS, so we cannot conclude if tRNS is superior or not. Second, we did not include additional groups targeting right and left PPC separately. Previous results^7^ found significant results on CT after both anodal left and anodal right PPC. However, we cannot rule out if there would have been differential effects on visual originality. Third, we did not include any measure of attention, memory or working memory, so we cannot rule out the possible mediating role of visual attention in the visual originality results obtained in the present study. Finally, although the highest electric-field strength was estimated to be under the PPC, adjacent areas may have also been receiving electrical stimulation so we cannot completely rule out the influence of adjacent areas on these results.

Future studies could directly compare if the stimulation of prefrontal brain regions increases fluency scores compared to a differential effect of PPC stimulation on originality scores. Regarding stimulation techniques, there is still much to research on the long-term effect of tRNS. Since most studies have used single session transcranial brain stimulation so far, future studies could also investigate if repetitive brain stimulation generates more pronounced and lasting effects. Additionally, future studies could also investigate if tRNS increases alpha activity or if the effects of 10 Hz tACS effects on alpha activity over PPC are related to creative enhancement.

## Material and methods

### Participants

A priori power analysis was conducted to determine the sample size using the G Power*3 software^82^. According to a previous study^7^, a sample size of 40 participants, 20 in each group, was enough to attain a large effect size (f = 0.54) to detect differences in a Compound Remote Associates task with 90% power and a 5% level of significance. Inclusion criteria included age of 18 years or above and both genders. Exclusion criteria included: (1) previous history of brain surgery; (2) being pregnant; (3) suffering from frequent or severe headaches or migraines; (4) previous history or presence of neurological disorder or injury (epileptic or convulsive seizure, brain stroke, severe brain injury; and (5) presence of any type of metallic implant in the brain.

Participants did not receive any financial compensation for participating in the study. Ethical approval was obtained from the Deusto University Ethics Committee (Ref: ETK-31/17-18).

All volunteers provided written informed consent to participate in the study and all experiments were performed in accordance with the tenets of the Declaration of Helsinki and they were free to withdraw at any time. Once the study was finished, participants allocated to the sham group were offered the opportunity to receive real brain stimulation.

### Measures

#### The Edinburgh Handedness Inventory

We assessed handedness with the Edinburgh Handedness Inventory^83^. In this inventory participants are asked to indicate their preference of hand use for 10 everyday activities. Scores ranged from 100 (perfectly right-handed) to − 100 (perfectly left-handed).

#### The Remote Associates Test (RAT)

The Spanish version of the original RAT^21^ was administered in order to assess CT. This activity involves identifying a solution word that is associated with three cue words. The solution can be related to the three cue words either semantically or by forming a compound word. Different forms of the test were employed for the baseline evaluation and after intervention. Each form included 30 items. Participants were given 2 min and 45 s. The number of correct responses was recorded and scores ranged from 0 to 30. Internal consistency was high (Cronbach´s alpha = 0.81).

#### The Torrance Test of Creative Thinking (TTCT)

The Picture Completion and the Unusual Uses (UU) subtests from the TTCT^65,84^ were administered in order to assess visual DT and verbal DT, respectively. Two minutes were given to perform each task. Different forms of the test (Form A and B) were administered for the baseline and after the stimulation. The Picture Completion task requires the participants to complete ten unfinished figures by adding additional elements. For this study three dimensions were measured: fluency, originality, and flexibility. These dimensions were scored using the Torrance Test of Creative Thinking scoring manual^84^. Fluency was measured by the total number of figures completed, awarding 1 point to each figure completed. Originality was based on the statistical infrequency of each response. Responses were classified as original (1 point) or unoriginal (0 points) according to a list that had been developed for each item by Torrance^84^ on the basis of normative data. Flexibility was defined as the number of different ideational categories produced in the pictures. For the correction of this dimension, each figure was classified according to the corresponding category, using the list of categories from the Spanish adaptation of the Torrance Test of Creative Thinking^85^. One point was given for each different category used. Fluency, originality and flexibility were converted into z scores based on the pooled group and a visual DT composite was obtained, showing a satisfactory internal consistency (Cronbach´s alpha = 0.84).

In the UU task, participants were asked to write as many unusual uses as possible for an item. In Form A of the test, the stimulus was Cardboard Boxes, while in Form B Tin Cans was used. Three dimensions were measured: Fluency, Originality, and Flexibility. Fluency was based upon the number of different unusual uses generated, assigning 1 point to each unusual use. Originality was based on the statistical unusualness of each response. This dimension was scored using the list from the manual^65,84^, giving 1 point for original or uncommon responses, and 0 points for unoriginal responses. Flexibility score was obtained from the number of different categories represented in the responses, using the list of categories from the manual^84^, awarding 1 point to each different category. Fluency, originality, and flexibility were used to obtain a verbal DT composite (Cronbach´s alpha = 0.85).

#### Questionnaire of adverse effects

After each session, subjects filled out a questionnaire to assess any perceived side effects, which consisted of 11 items (including headache, throat sore, scalp pain, skin tingling, skin itching, skin burning sensation, redness of the skin, numbness, concentration problems, mood change and phosphenes).

### Transcranial stimulation protocol

**Figure 1:**
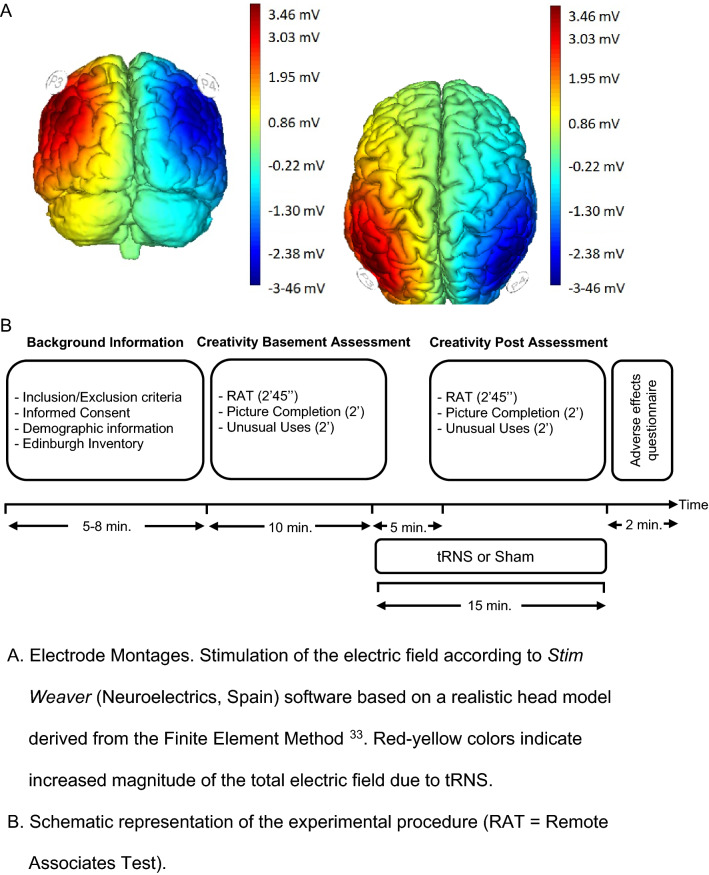
Study design and transcranial random noise stimulation (tRNS) montage.

In the tRNS and sham groups, the electrodes were placed over the left and right PPC (P3 and P4, respectively). TRNS group received 1.5 mA (tRNS 100–500 Hz) via two saline-soaked (5 ml per sponge), 16 cm2 (8 × 8 cm2) circular sponges. They were attached under designated electrode positions (P3, P4) using a wireless neoprene cap that followed the International 10–20 system. Figure 1 (part A) shows the simulated electric field of this tRNS group (based on Stim Weaver) using the finite element model^86^. TRNS was applied with a light, battery-operated device (Neuroelectrics Inc., Barcelona) attached to the back of the neoprene cap, delivering electrical current for 15 min with additional ramp-up and ramp-down phases of 30 s. In the sham condition, current was applied using a 30 s ramp up followed 15 min after by a 30 s ramp-down of activity. The impedance of the electrodes was checked before and during tRNS application to guarantee that it was below 10 kΩ.

### Procedure

The study had a double-blind, sham-controlled, parallel-group design. The participants were randomly allocated to either the tRNS or the sham group (see Fig. 1, Part A). Assignment was conducted based on a computer-generated randomization (randomizer.org). All raters (JP, AS, and AA) were blind to treatment condition.

Figure 1 (part B) shows the study design and procedure. After signing the consent form, participants reported sociodemographic information along with hours of sleep, tobacco consumption, and stimulant drinks ingested before the session. Afterwards, the Edinburgh Handedness Inventory was administered. Baseline creativity assessment was carried out before starting stimulation (tRNS or sham). They have 2 min and 45 s to complete the RAT, 2 min for UU and 2 min Figure Completion.

Five minutes after the beginning of stimulation, they started performing the parallel versions of RAT, UU and Figure completion tests with the same time limitations. The order of RAT, UU, and Figure Completion versions were counterbalanced. Afterwards, participants completed the adverse effects questionnaire. Finally, in order to examine the blinding efficacy, participants were asked to answer the following question: “Please, tell us if you think you were receiving real stimulation, no stimulation (placebo) or you do not know”.

### Statistical analyses

Categorical data were analyzed with the χ^2^ test. Baseline characteristics were compared using ANOVA. Analyses of covariance (ANCOVA) were performed independently for CT, visual DT and verbal DT with the post test scores as dependent variable and the baseline scores as covariate. Effect size (${\mathrm{n}}_{\mathrm{p}}^{2}$) was reported and according to Cohen^87^, an effect size of 0.01 is considered small, 0.06 medium, and 0.14 large. IBM SPSS Statistics Version 25 was used for all statistical analyses. The significance level was set at 0.05. All tests were two-tailed.

## Data Availability

The datasets generated during and/or analyzed during the current study are available from the corresponding author on reasonable request.
